# Structure-Based Redesign of a Self-Sufficient Flavin-Containing Monooxygenase towards Indigo Production

**DOI:** 10.3390/ijms20246148

**Published:** 2019-12-05

**Authors:** Nikola Lončar, Hugo L. van Beek, Marco W. Fraaije

**Affiliations:** 1 GECCO Biotech, 9747 AG Groningen, The Netherlands; n.loncar@gecco-biotech.com; 2 Molecular Enzymology group, University of Groningen, 9747 AG Groningen, The Netherlands; h.l.van.beek@rug.nl

**Keywords:** indigo, MISO library, flavin, monooxygenase, FMO

## Abstract

Indigo is currently produced by a century-old petrochemical-based process, therefore it is highly attractive to develop a more environmentally benign and efficient biotechnological process to produce this timeless dye. Flavin-containing monooxygenases (FMOs) are able to oxidize a wide variety of substrates. In this paper we show that the bacterial mFMO can be adapted to improve its ability to convert indole into indigo. The improvement was achieved by a combination of computational and structure-inspired enzyme redesign. We showed that the thermostability and the *k*_cat_ for indole could be improved 1.5-fold by screening a relatively small number of enzyme mutants. This project not only resulted in an improved biocatalyst but also provided an improved understanding of the structural elements that determine the activity of mFMO and provides hints for further improvement of the monooxygenase as biocatalyst.

## 1. Introduction

Indigo is one of the oldest dyes known to mankind. It was used in ancient Egypt to dye clothes as early as 2300 B.C. [1]. Originally indigo was produced from plants such as *Indigofera spp*. and *Polygonum tinctorum* [1]. The chemical process that was developed in 1883 by Adolph von Baeyer is still used in the 21st century [2]. One of the advantages of synthetic indigo is the purity, already reaching >90% in 1900 [3], while natural indigo from *Indigofera tinctoria* is obtained with a purity of between 20% and 90% [3]. Enzymatic oxidation of indole could result in indigo of a higher purity as enzymes are typically highly selective. The current annual production of indigo is estimated at 80,000 tons per year [4] which is mainly used as a dye in the textile industry to produce blue jeans, applying 3–12 g of indigo to dye a pair of jeans [4]. 

The demand for a cleaner process triggered microbiological research concerning indigo production. In 1983 the first naphthalene oxidation genes were expressed in *Escherichia coli* allowing indigo formation in the presence of tryptophan or indole [5], and which was further exploited [6]. Metabolic engineering was used to increase in vivo indole concentrations by modifying the tryptophan metabolism pathway combined with tuning expression of naphthalene dioxygenase (NDO) from *Pseudomonas putida* [7]. However, more extensive metabolic engineering is needed to adjust the overall metabolism of *E. coli* for indigo production [7]. Another disadvantage of NDOs is their susceptibility to inactivation [8]. In later studies it was demonstrated that various other enzymes can also be used for indigoid dye production, such as P450 monooxygenases [9,10] and styrene monooxygenases [11,12]. Another promising alternative enzyme was described by Choi et al. [13] who discovered the flavin-containing monooxygenase from *Methylophaga* sp. (mFMO) that is able to oxidize indole, resulting in the formation of indigo after non-enzymatic dimerization. Han et al. subsequently carried out optimization of indigo production by whole-cells containing mFMO yielding 920 mg/L of indigo [14]. Alfieri et al. (2008) solved the crystal structure of mFMO and described the moonlighting role of NADP in the structure of mFMO [15]. 

Wild-type mFMO is active towards indole but its substrate recognition and turnover rates are rather poor, especially when compared to other substrates such as trimethylamine [15] that show a *k*_cat_ that is more than eight-fold higher. This work aimed at improving the catalytic performance of the monooxygenase by structure-based enzyme redesign. To obtain an improved monooxygenase, first mutations were identified that improve the thermostability of mFMO using computational predictions [16,17]. Then, residues that form the substrate binding pocket have been replaced using site-directed mutagenesis. Mutations that led to improved catalytic efficiency were combined using multichange isothermal mutagenesis (MISO) [18] to create an mFMO mutant that is more effective in oxidizing indole. These experiments have resulted in an improved biocatalyst and provide more insight into the structural elements that tune the activity of mFMO. Together with more thorough characterization of this enzyme, this study provides a basis for further improvement of this monooxygenase as biocatalyst.

## 2. Results

Engineering a thermostable variant of mFMO—engineering an active site of the enzyme often results in mutants with decreased stability. In an ideal scenario, one would like to have an as stable as possible starting enzyme. mFMO falls into the category of moderately stable enzymes with an apparent melting temperature of 43.3 °C. The recently developed FRESCO protocol [16,17] is an effective tool for the stabilization of enzymes, including flavin-containing enzymes [19]. Through this computational protocol, mutations are predicted that should render the target protein more (thermo) stable. It involves the fully automated in silico generation of all possible single mutants and the respective folding energy calculations of the modeled structures of these mutants. In a next step, mutant structures that appear more stable according to these calculations are subjected to molecular dynamics simulations to filter out mutations that are not compatible with the structural dynamics. In a final visual inspection round, typically 100–200 mutations are selected that have the best energy scores. As a first step towards transforming mFMO into a robust biocatalyst, the FRESCO analysis was performed. Based on this, 140 mutant proteins were expressed and purified. Analysis of all these mutants resulted in the identification of 16 mutant enzymes that show an increase of apparent melting temperature of >1 °C (Table S1). Combining two mutations at the N-terminus (M15L and S23A) resulted in a 3 °C increase in apparent melting temperature. Both M15L and S23A are far from the active site (>17 Å) and we observed no significant effect on the kinetic parameters of the enzyme. Unfortunately, adding more stabilizing mutations did not contribute to a higher thermostability. In the rest of the manuscript, *mFMO is used to indicate the use of this M15L/S23A double mutant which is a more stable starting point for the protein engineering campaign aimed at improving its catalytic performance on indole. 

**Figure 1:**
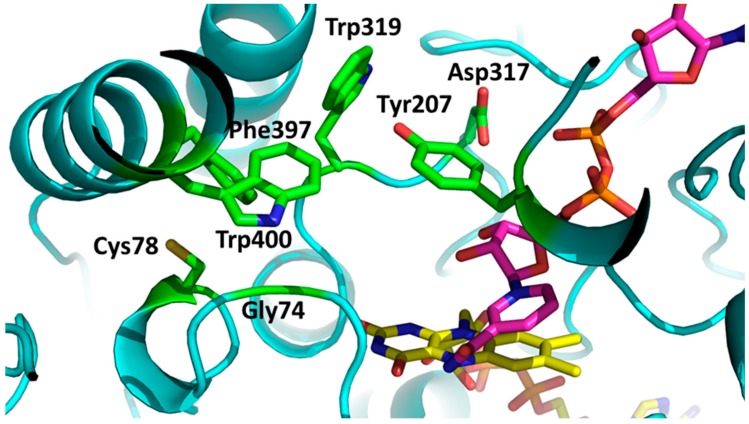
Active site of flavin-containing monooxygenase from *Methylophaga* sp. (mFMO). The residues forming a pocket next to the reactive moiety of the flavin cofactor are labeled and highlighted in green. The FAD cofactor is in yellow and the bound NADP^+^ is in magenta. (PDB:2VQ7).

Engineering mFMO for improved catalytic performance with indole—in order to improve the activity of mFMO on indole we focused on the active site of the enzyme. When inspecting the crystal structure of mFMO that contains both cofactors, FAD and NADP^+^, a cavity close to the reactive moiety of the flavin cofactor can be identified [15]. The residues that form the entrance and shape of this cavity are highlighted in Figure 1 and listed in Table 1). Several mutant enzymes targeting the active site residues and combinations thereof have been expressed, purified, and evaluated using kinetic measurements.

In this first set of mutants we observed that they all retained activity (Table 2). Mutations on two positions resulted in beneficial effects: C78I and C78V increased the *k*_cat_ and C78A and Y207W decreased the K_M_ (although these mutations also decreased the *k*_cat_). While mutating Trp319 did not directly lead to improvements, we remained interested in this position because it seems to have a major role in substrate binding. Based on these initial results we designed a MISO library. MISO allows the generation of a mutant library in which specific residues are allowed to mutate into a pre-designed set of alternative residues. Because mFMO activity could only be measured reliably using purified enzyme, we limited the size of the library to 48 possible mutations. For Cys78, we allowed the wild-type residue cysteine, the beneficial mutations to isoleucine, valine, and phenylalanine. For Tyr207, we allowed the wild-type residue tyrosine, the tryptophan, that was found to increase the affinity for indole, and asparagine. For Trp319, we allowed the wild-type tryptophan and phenylalanine, alanine, and asparagine. Instead of over screening by purifying (many) more mutants than the library size to get good coverage, we sequenced 190 clones first. In this set of clones, we obtained 44 of the 48 expected mutants. The respective mutant enzymes were obtained by growing cells at 15 mL scale and purification using 96-well plates [16]. After determining the concentration based on the absorbance at 441 nm, we performed a kinetic analysis of the mutants. We measured the uncoupling rate (the rate of NADPH consumption in the absence of substrate) and the activity with indole or TMA. A complete overview of the screened activities with indole and TMA and uncoupling rates can be found in the Supplementary Materials (Table S2).

Screening of the MISO library led to the discovery of several mutants with increased activity compared to *mFMO (Table 2). The C78I mutant was the most active variant in the library, and also the C78V and Y207W/W319A mutants were identified as improved variants. Several other patterns were observed in the data obtained from screening the MISO library. Asparagine at position 207 leads to enzymes with a high uncoupling rate, still consuming NADPH but producing hydrogen peroxide instead of a hydroxylated product. A phenylalanine on position 78 leads to low activity. Several mutants, also some that only showed rates similar to *mFMO, were characterized in more detail (Table 2). Besides mutants showing a clear improvement in *k*_cat_ (C78I, C78V, C78I/Y207W/W319A) we also found mutants containing both the Y207W and W319A mutation to be active. These variants show behavior similar to the *mFMO when both mutations are introduced together. The Y207W mutation by itself reduces activity (but improved K_M_) and the W319A by itself has a negative effect on both *k*_cat_ and K_M_. This effect was observed in combination with the wild-type Cys78, as well as in combination with C78I and C78V.

**Figure 2:**
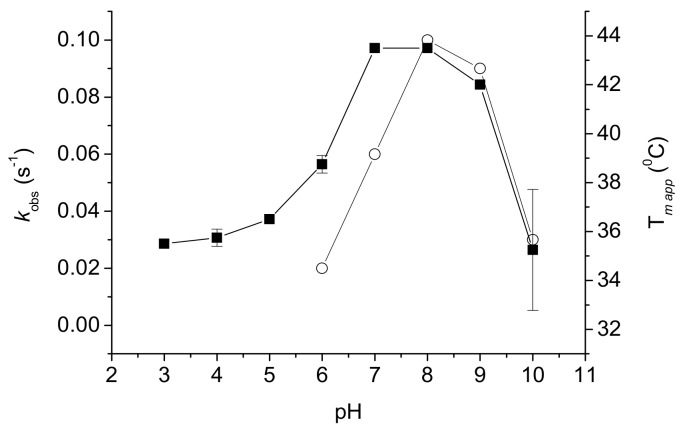
The effect of pH on the activity and stability of PTDH-*mFMO. (○)-activity (s^−1^), (■)-stability as melting temperatures (°C).

Exploring conditions for mFMO-catalyzed indigo production—in addition to the engineering of the enzyme, we determined the optimal conditions for the enzyme. The pH optimum for activity was determined to be at pH 7.0–8.0, with most of the activity retained at pH 9 (Figure 2). The activity drops significantly at both lower and higher pH values. Interestingly, the stability of *mFMO shows a sharper pH optimum, with the highest thermostability at pH 8 (Figure 2). While at pH 9 the thermostability is still relatively high, it quickly drops at pH < 7 and pH > 9.

The enzyme is optimally active at 250 mM NaCl, with an activity increase of more than 25% compared to the enzyme in the absence of NaCl (Figure S1). The enzyme is more stable with higher concentrations of NaCl. Glycerol has a slight stabilizing effect but no effect on activity (Figure S2A). DMSO up to 4% halves the activity while the enzyme remains quite stable (Figures S2B and S3B). Methanol also has a detrimental effect on stability, and dioxane is poorly tolerated by the enzyme (Figure S3B). Interestingly, NADP^+^ as additive revealed an optimum for thermostability. In the range of 10–50 µM NADP^+^, *mFMO is slightly stabilized, while higher concentrations lead to a lower thermostability (Figure S3A).

A layer of indigo-paste has to be scraped of the cell pellet before further processing and purification of *mFMO. Conversions with purified enzyme and cell-free extracts were done to quantify the indigo yield, as well as the purity with regards to the contaminant indirubin. The stability and activity measurements showed pH 8 is the optimum for performing conversions. NaCl and glycerol seem to have both a stabilizing effect as well as a positive effect on enzymatic activity. However, with the aim of developing a cost-effective process, no additives were used in the conversions. A reaction mixture containing only the essential components (see materials and methods) was used. To reduce the costs related to the use of NADPH, a catalytic amount of NADP^+^ is used in combination with the fused cofactor regeneration system PTDH. Conversions were performed using either purified PTDH-*mFMO or PTDH-*mFMO cell free extract (CFE) (the use of CFE would reduce the cost of a process). This resulted in significant differences in both indigo yield and purity (Table 3). Indigo produced using CFE was found to be 94% pure while the use of purified enzyme resulted in >99% pure indigo. Synthetic indigo is known to reach purities of over 90% [20], with most available synthetic produced indigo having a purity of 94%. Purified PTDH-*mFMO seems to reach even higher purity levels, since the amount of indirubin is not detectable when measured with HPLC. We set up conversions in a such a way that the amount of enzyme would be limiting and a conversion of only 23% or 14%–18% was found for the purified enzyme or the CFE, respectively. The total turnover number was determined to be 5700, showing that there is some inactivation of the enzyme during the 24 h reaction time. 

## 3. Discussion

Already the wild-type mFMO expressing *E. coli* cultures develop an intense blue color clearly demonstrating the potential for indigo production. When the enzyme was studied in more detail, it was obvious that the enzyme prefers other substrates, for example trimethylamine over indole. This leaves room to improve the performance of mFMO on indole by engineering a variant with a higher affinity and catalytic rate by optimizing the fit of this molecule in the active site. In this study, we show that it is possible to improve the stability and catalytic performance on indole of this monooxygenase. The higher thermostability was achieved using the FRESCO protocol, which is a structure-based computational methodology which predicts mutations that improve thermostability of enzymes. Based on the structure of the enzyme we also generated and tested several mutants for improved catalytic performance. Small effects are expected from the second shell mutations, so the rather large effect of C78I is surprising. Based on the initial results with single mutants, a MISO library was built to further explore a small part of the sequence space. By first sequencing the library and subsequently only purifying unique mutants we were able to efficiently gather information about almost every mutant in the library. Unfortunately, this did not lead to mutants that are faster than the initially created C78I. By screening almost the entire library we obtained valuable knowledge on the mutability of the mFMO active site; we thoroughly probed Cys78, a very influential secondary-shell residue and found a synergistic mutation where a tryptophan is swapped with another aromatic residue. We also identified a mutation, Y207W, that results in a higher affinity for indole while also affecting the activity. The effect of the Y207W mutation on the catalytic performance of mFMO is in line with a previously reported Y207S mFMO mutant [21]. Cho et al. have shown that this specific mutation nearly abolishes all indole oxygenation activity of mFMO. Inspection of the available structures of mFMO suggests that Tyr207 is important in positioning the nicotinamide cofactor. Previous work has shown that mFMO and structurally related flavoprotein monooxygenases require a tightly bound nicotinamide cofactor, even after it has transferred a hydride to the flavin cofactor, for catalyzing oxygenations [22,23,24,25,26]. Thus, proper positioning of the nicotinamide cofactor in the NADP^+^-complexed mFMO is important for indole oxidation. Tyr207 also points towards a cavity that can accommodate substrates in the NADP^+^-bound mFMO structure. As a consequence, replacing Y207 can affect the activity and affinity for indole indirectly, through altering the position of the nicotinamide cofactor, or by directly influencing the substrate binding pocket.

We showed that the activity of *mFMO can be improved. The comprehensive results from the MISO library screening give a solid basis on which to design larger libraries. Some mutations primarily increased the *k*_cat_, which is beneficial for any kind of indole conversion process, but depending on the used concentration of indole a lower K_M_, which was also found for some mutants, might be equally desirable. 

The thermostable *mFMO variant was used for conversions, both as purified enzyme and as cell-free extract. The relatively low conversion is primarily caused by the low enzyme loading: The measured total turnover number, 5700, could even be improved by applying a mutant enzyme or better conditions. 

Conditions such as pH, solvents and salt concentrations were explored for the *mFMO. The optimum pH was eight or nine, with the activity decreasing more quickly than stability at lower pH. Solvents in general are detrimental for the enzyme, precluding cosolvent use. 

## 4. Materials and Methods 

### 4.1. Chemicals and Reagents 

Indigo and indole were purchased from Sigma Aldrich (St. Louis, MO, USA). NADP^+^ and NADPH were purchased from Oriental yeast Co (Tokyo, Japan). Other chemicals were analytical grade and obtained from either Sigma-Aldrich or Merck (Burlington, MA, USA). 

### 4.2. Strains, Plasmids and Growth Conditions

Recombinant PTDH-mFMO was overexpressed and purified following the previously described procedure [27]. Briefly, *E. coli* NEB10β cells containing the pCRE3-mFMO plasmid grown overnight were diluted 100-fold in TBamp medium and induced directly with 0.02% arabinose. After incubation at 24 °C and 135 rpm (5 cm amplitude) for 48 h the cells were harvested (4 °C, 4000 × *g* for 15 min). The cell pellet was resuspended in 50 mM KPi buffer pH 7.5, disrupted by sonication, and the cell free extract was obtained by centrifugation at 4 °C 18,500 × *g* for 45 min. CFE was stored in 50 mM KPi buffer pH 8 containing 10% glycerol at −20°C after being frozen in liquid nitrogen. Purification of PTDH-mFMO was carried out on 5 mL HiTrap Ni-Sepharose column using ÄKTA system (Chicago, IL, USA). Concentrations of purified PTDH-mFMO was determined by using extinction coefficient of 14.4 mM^−1^ cm^−1^ at 441 nm.

### 4.3. Steady-State Kinetic Analyses

Kinetic assays were performed in triplicate using a BioTek synergy MX (Winooski, VT, USA) or BioTek synergy H1 platereader. Enzyme was used at 0.5 or 0.05 µM depending on the substrate. Different concentrations of substrate were used, up to 1.6 mM indole and 0.20 mM TMA. For indole, the solubility of the substrate was a limiting factor, for TMA the K_M_ values require a lower concentration of the substrate and a lower concentration of enzyme. Reactions were started by adding 100 µL NADPH to 100 µL enzyme-substrate mix. The depletion of NADPH was followed over time by measuring the 340 nm absorbance, using an extinction coefficient for NADPH of 6.22 mM^−1^ cm^−1^. No correction was made based on the uncoupling rate in the absence of substrate, because the *k*_cat_ and K_M_ values were not significantly affected by this correction. The standard deviations for the determined kinetic parameters were typically <20%.

### 4.4. pH Optimum

Determination of the pH optimum of PTDH-mFMO activity was performed by using abovementioned procedure with the difference of using Britton Robinson buffer in pH range 2.0–12.0. Final concentrations in the 1 mL reaction mixture were 4 mM indole, 0.4 µM PTDH-mFMO and 0.2 mM NADPH.

### 4.5. ThermoFAD

The ThermoFAD method [28] was used to determine the apparent melting points of the PTDH-mFMO in different pH conditions or in presence of additives. Using an RT-PCR machine (CFX96-Touch, Bio-Rad, Hercules, CA, USA) the fluorescence of the FAD cofactor was monitored using a 450–490 excitation filter and a 515–530 nm emission filter, typically used for SYBR Green based RT-PCR. The temperature was increased with 0.5 °C per step, starting at 25 °C and ending at 90 °C, using a 10 s holding time at each step. The maximum of the first derivative of the observed flavin fluorescence was taken as the apparent melting temperature. 20 µL of the enzyme solution contain 10 µM PTDH-mFMO and the additive (solvent or buffer of appropriate pH) was put into a 96 wells PCR plate and covered with transparent cover, then ThermoFAD was ran using the BioRad CFX manager. The used additives were: Glycerol in concentrations of 0%, 1%, 5%, 10%, and 20%; NaCl in concentrations of 0, 50, 135, 250, and 500 mM; and NADP^+^ in concentrations of 0, 1, 10, 50, and 100 µM. The used solvents were DMSO, methanol, and 1,4-dioxane all of which were used in concentrations of 0%, 1%, 5%, 10%, and 20%.

### 4.6. Indole Conversion by Purified PTDH-mFMO and Cell Free Extract (CFE)

Reactions of PTDH-mFMO with indole were set up to determine the efficiency of the indigo production. For this, final concentrations of 0.15 mM NADP^+^, 0.4 µM FAD, 0.4 µM PTDH-mFMO, 20 mM Na-phosphite, 10 µL catalase (300 U), and 10 mM indole were used in 1 L 50 mM KPi buffer pH 8.0 in 2 L Erlenmeyer flask incubated at room temperature and stirred with magnetic stirrer. After 24 h the reaction mixture was centrifuged at 4000 × g to pellet the formed indigo. The indigo pellets were washed twice in demineralized water and once in 70% ethanol and dried until constant weight. Samples were dissolved in DMSO, diluted in methanol, and analyzed by HPLC. Conversions (1 L) were also performed with cell free extract containing PTDH-mFMO in a similar fashion as with the purified enzyme with some slight modifications. The reaction mixture consisted of: An estimated amount of 0.4 µM PTDH-mFMO in CFE, 1 mM Na-phosphite, 10 µL catalase (300 U), and 10 mM indole.

### 4.7. HPLC Analysis for Indigo Purity Measurements

Analyses were performed at 25 °C on an Alltima HP C18 column (3 μm, 4.6 by 150 mm I.D.; Grace, Reading, UK), equipped with a guard column. The mobile phase consisted of water containing 0.1% formic acid (eluent A) and acetonitrile (eluent B). The gradient program used was as follows: 10% B 2 min, 10% to 100% B (30 min), 100% B isocratic (5 min), and 100% to 10% B (3 min), followed by re-equilibration for 7 min. The flow rate was 0.5 mL/min and the injection volume were 20 µL. Commercially available indigo and indirubin were used as reference. Detector was set at 600 nm, since indigo has a maximal absorption at 610 nm whereas indirubin has a maximal absorption at 546 nm, as determined spectrophotometrically using standards. 

## 5. Conclusions

Using rationally designed mutants we have shown it is possible to improve the flavin-containing monooxygenase mFMO on indole as substrate. A MISO library was used to expand the sequence space that was explored for improved activity, identifying further mutants with enhanced activity. We also showed that the wild-type enzyme already shows sufficient activity to perform conversions of indole to indigo. The set of improved mutants are good starting points for further engineering of the enzyme.

## Figures and Tables

**Table 1:** Amino acid residues expected to affect substrate binding in mFMO.

Position	Comments
Gly74	A sidechain at this position could have interactions with the substrate. Despite being a pre-Pro residue, the phi-psi angles (−130,175°) will also fit non-Glycine residues.
Cys78	Its mutation could fill up a cavity in the active site making binding of indole more productive.
Tyr207	Forms part of entrance to the substrate binding cavity.
Asp317	Forms part of entrance to the substrate binding cavity.
Trp319	Limits the size of the substrate binding cavity.
Phe397	Limits the size of the substrate binding cavity.
Trp400	Limits the size of the substrate binding cavity.

**Table 2:** Kinetic parameters determined for *mFMO and mutants obtained from initial rational design and the multichange isothermal mutagenesis (MISO) library.

	Indole	
**Variant**	***k*_cat_ (s^−1^)**	**K_M_ (mM)**
WT	0.85	0.4
Site-directed mutants		
C78I	1.28	0.8
C78V	1.04	0.7
C78L	0.70	0.8
C78A	0.79	0.4
W319A	0.42	0.9
W319F	0.64	0.4
Y207W	0.31	0.1
MISO mutants		
Y207W/W319A	0.80	0.8
C78I/Y207W/W319A	0.93	0.8

**Table 3:** Indigo yield after 24 h conversion of 10 mM indole with purified enzyme or two different batches of cell free extract (CFE).

	Indigo Yield (g/L)	Conversion (%)	Purity (%)	TTN
PTDH-mFMO	0.30	23	>99	5700
PTDH-mFMO CFE 1	0.24	18	94	n.a.
PTDH-mFMO CFE 2	0.18	14	94	n.a.

Conversion is calculated as the recovered amount of indigo compared to the theoretical yield. Indigo purity is reported as the percentage of indigo of the total amount of indigo and indirubin as analyzed by HPLC. The total turnover number (TTN) for CFE could not be determined as the exact amount of enzyme is not known. n.a.: not applicable.

## Supplementary Materials

Supplementary materials can be found at https://www.mdpi.com/1422-0067/20/24/6148/s1.

## Abbreviations

FRESCO	Framework for Rapid Enzyme Stabilization by Computational libraries
MISO	Multichange ISOthermal mutagenesis
