# Supplementary material for: Structure-Based Redesign of a Self-Sufficient Flavin-Containing Monooxygenase towards Indigo Production
Source: Int J Mol Sci. 2019 Dec 5;20(24):6148. doi: 10.3390/ijms20246148 (PMC6940849; doi:10.3390/ijms20246148)
Supplement: Supplementary file 1 [file ijms-20-06148-s001.pdf]

Supplementary Information

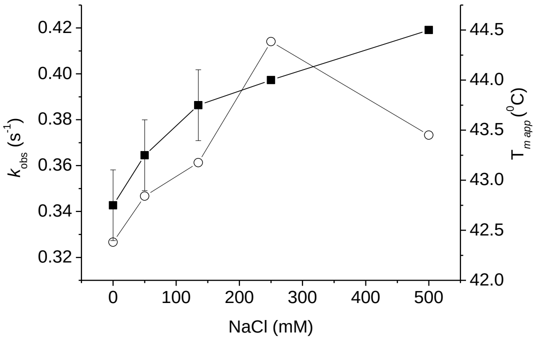

Fig. S1 The effect of different concentrations of NaCl on the activity and stability of PTDH-mFMO. (○)-activity (s^{-1}), (■)-stability (°C).

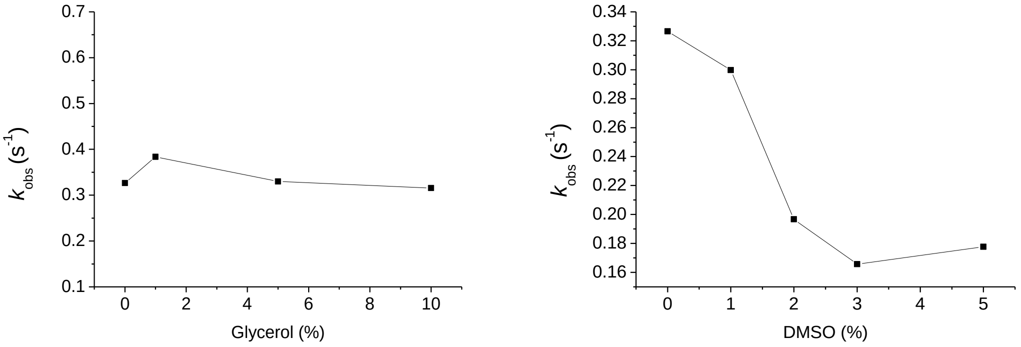

Fig. S2 The effect of glycerol (A) and DMSO (B) on activity of PTDH-mFMO.

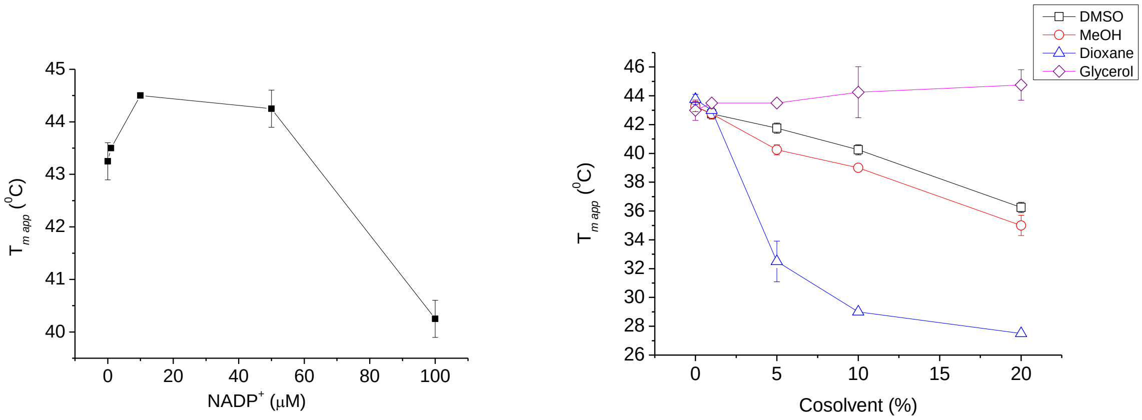

Fig. S3 The effect of $NADP^+$ (A) and different cosolvents (B) on the apparent melting temperature (Tm app) of PTDH-mFMO.

Table S1. List of beneficial mutations as a result of FRESCO study on mFMO with given increase in apparent melting temperature according to the ThermoFAD assay.

Position	ΔT*m app* (^{o}C)
M15L	2.9
S23A	2
E130K	1.4
V200I	1.4
N254Y	1.4
I275L	1.5
W319F	1.5
Q377K	2.25
N378T	1.5
H402A/ H402K	2
S415R	1.5
H428P	2
H428M	1.5
E441Q	1.5
A442Y	1.5

Table S2. MISO screening data summary. [a] mutants designated by the one letter code for positions 78, 207 and 319. [b] observed rate (s^{-1}) with 1.6 mM indole, average for two technical duplicates for mutants or the average of two technical duplicates for three separate cultures for the wild-type. [c] observed rate (s^{-1}) with 0.2 mM trimethylamine (TMA), single datapoint. [d] uncoupling rate (s^{-1}) in the absence of substrate. [e] concentration of enzyme based on FAD absorption (μM) as an indication for purification yield. [f] reason for rejection for further experiments; for selected mutants the observed rate (s^{-1}) with 1.6 mM indole (column 2) is listed.

Mutant[a]	indole[b]	TMA[c]	uncoupling[d]	yield[e]	selection[f]
**C (WT) - Y (WT) - W (WT)**	0.12	0.74	0.05	26	0.115
C (WT) - N - A	0.06	0.06	0.06	16	significantly slower than WT
C (WT) - N - F	0.10	0.12	0.11	16	relatively high uncoupling
C (WT) - N - N	0.14	0.17	0.16	17	relatively high uncoupling
C (WT) - N - W (WT)	0.15	0.18	0.14	18	relatively high uncoupling
C (WT) - W - A	0.18	0.45	0.06	23	0.177
C (WT) - W - C	0.05	0.25	0.04	23	significantly slower than WT
C (WT) - W - F	0.03	0.31	0.05	25	significantly slower than WT
C (WT) - W - N	0.07	0.36	0.06	22	significantly slower than WT
C (WT) - W - W (WT)	0.06	0.35	0.07	22	significantly slower than WT
C (WT) - Y (WT) - A	0.09	0.63	0.04	25	significantly slower than WT
C (WT) - Y (WT) - F	0.10	0.68	0.04	24	0.103
C (WT) - Y (WT) - N	0.04	0.48	0.03	23	significantly slower than WT
F - N - A	0.05	0.04	0.04	18	significantly slower than WT
F - N - F	0.08	0.06	0.05	18	significantly slower than WT
F - N - N	0.03	0.03	0.02	15	significantly slower than WT
F - N - W (WT)	0.08	0.07	0.06	20	significantly slower than WT
F - W - F	0.07	0.10	0.07	26	significantly slower than WT
F - W - N	0.05	0.05	0.04	18	significantly slower than WT
F - W - W (WT)	0.05	0.08	0.05	20	significantly slower than WT
F - Y (WT) - F	0.08	0.18	0.07	19	significantly slower than WT
F - Y (WT) - N	0.06	0.06	0.05	18	significantly slower than WT
I - N - A	0.17	0.16	0.16	18	relatively high uncoupling
I - N - F	0.18	0.18	0.19	21	relatively high uncoupling
I - N - N	0.14	0.15	0.16	20	relatively high uncoupling
I - N - W (WT)	0.19	0.20	0.21	19	relatively high uncoupling
I - W - A	0.13	0.32	0.05	21	0.126

I - W - F	0.09	0.36	0.06	22	significantly slower than WT
I - W - N	0.04	0.15	0.03	21	significantly slower than WT
I - W - W (WT)	0.06	0.23	0.06	22	significantly slower than WT
I - Y (WT) - A	0.09	0.38	0.08	22	significantly slower than WT
I - Y (WT) - F	0.13	0.61	0.04	22	0.134
I - Y (WT) - N	0.04	0.35	0.03	21	significantly slower than WT
I - Y (WT) - W (WT)	0.19	0.56	0.05	24	0.190
V - N - A	0.13	0.26	0.12	22	relatively high uncoupling
V - N - F	0.11	0.11	0.14	21	relatively high uncoupling
V - N - N	-0.03	-0.01	0.01	16	significantly slower than WT
V - N - W (WT)	0.15	0.14	0.16	19	relatively high uncoupling
V - W - A	0.14	0.25	0.07	24	0.141
V - W - F	0.08	0.36	0.06	23	significantly slower than WT
V - W - N	0.08	0.27	0.06	26	significantly slower than WT
V - W - W (WT)	0.06	0.25	0.05	26	significantly slower than WT
V - Y (WT) - A	0.11	0.54	0.04	26	0.106
V - Y (WT) - F	0.10	0.40	0.03	24	0.104
V - Y (WT) - N	0.10	0.25	0.10	24	relatively high uncoupling
V - Y (WT) - W (WT)	0.24	0.68	0.04	20	0.235
